# 12 years on – Is the NLM medical text indexer still useful and relevant?

**DOI:** 10.1186/s13326-017-0113-5

**Published:** 2017-02-23

**Authors:** James Mork, Alan Aronson, Dina Demner-Fushman

**Affiliations:** US National Library of Medicine, 8600 Rockville Pike, Bethesda, USA

**Keywords:** Indexing methods, Text categorization, MeSH, MEDLINE, Machine learning, BioASQ

## Abstract

**Background:**

Facing a growing workload and dwindling resources, the US National Library of Medicine (NLM) created the Indexing Initiative project in 1996. This cross-library team’s mission is to explore indexing methodologies for ensuring quality and currency of NLM document collections. The NLM Medical Text Indexer (MTI) is the main product of this project and has been providing automated indexing recommendations since 2002. After all of this time, the questions arise whether MTI is still useful and relevant.

**Methods:**

To answer the question about MTI usefulness, we track a wide variety of statistics related to how frequently MEDLINE indexers refer to MTI recommendations, how well MTI performs against human indexing, and how often MTI is used. To answer the question of MTI relevancy compared to other available tools, we have participated in the 2013 and 2014 BioASQ Challenges. The BioASQ Challenges have provided us with an unbiased comparison between the MTI system and other systems performing the same task.

**Results:**

Indexers have continually increased their use of MTI recommendations over the years from 15.75% of the articles they index in 2002 to 62.44% in 2014 showing that the indexers find MTI to be increasingly useful. The MTI performance statistics show significant improvement in Precision (**+0.2992**) and F_1_ (**+0.1997**) with modest gains in Recall (**+0.0454**) over the years. MTI consistency is comparable to the available indexer consistency studies. MTI performed well in both of the BioASQ Challenges ranking within the top tier teams.

**Conclusions:**

Based on our findings, yes, MTI is still relevant and useful, and needs to be improved and expanded. The BioASQ Challenge results have shown that we need to incorporate more machine learning into MTI while still retaining the indexing rules that have earned MTI the indexers’ trust over the years. We also need to expand MTI through the use of full text, when and where it is available, to provide coverage of indexing terms that are typically only found in the full text. The role of MTI at NLM is also expanding into new areas, further reinforcing the idea that MTI is increasingly useful and relevant.

## Background

For more than 150 years, the US National Library of Medicine (NLM) has provided access to the biomedical literature through the analytical efforts of human indexers. Since 1966, access has been provided in the form of electronically searchable document surrogates consisting of bibliographic citations, descriptors assigned by indexers from the Medical Subject Headings (MeSH®;) [1] controlled vocabulary and, since 1975, author abstracts for many citations.

The MEDLINE®;/PubMed®; database (MEDLINE) contains over 23 million citations. It currently grows at the rate of about 760,000 citations per year and covers over 5600 international biomedical journals in 36 languages. Human indexing consists of reviewing the full text of each article, rather than just the abstract or summary, and assigning Descriptors from the MeSH vocabulary that represent the central concepts as well as every other topic that is discussed to a significant extent.

### MeSH vocabulary

In the 2015 MeSH vocabulary, there are 27,455 Descriptors, which are often referred to as MeSH Headings (e.g., *Lung*). The scope of main heading descriptors may be refined further by selections from a collection of 83 topical MeSH Subheadings which are also known as Qualifiers (e.g., *Lung/abnormalities* means that the article is about the *abnormalities* associated with the *Lung* more than the *Lung* itself). In addition the vocabulary contains 225,067 Supplementary Concept Records (formerly called Supplementary Chemicals) consisting of chemicals, drugs, proteins, and diseases. Each Supplementary Concept Record is linked to one or more MeSH Heading via their “Heading Mapped to” entries (e.g., *Achondroplastic dwarfism* is linked to MeSH Main Heading *Achondroplasia*). MeSH Check Tags are a special type of MeSH Heading that are required to be included for each article and cover species, sex, human age groups, and pregnancy (e.g., *Male*) [2].

### Impact of MEDLINE indexing

Since 1990, there has been a steady and sizeable increase in the number of articles indexed for MEDLINE, because of both an increase in the number of in-scope articles in journals that are already being indexed and, to a lesser extent an increase in the number of indexed journals. NLM expects to index over one million articles annually within a few years.

MEDLINE Indexing has been used by librarians and researchers from its inception in 1879 by John Shaw Billings [3] and is currently used by an even larger community through PubMed [4]. PubMed uses the MEDLINE Indexing as part of their Automatic Term Mapping query expansion [5] and through their result filtering which depends on MEDLINE Indexing for determining species, sex, and ages [6]. Other recent examples of specific uses of MEDLINE Indexing include the results of TREC Genomics track (2003 – 2007) [7] and TREC Clinical Decision Support track (2014 - ongoing) [8] which show that the judicial use of manual MEDLINE indexing in faceted retrieval or for query expansion leads to at least moderate, and in some cases to significant improvements in Mean Average Precision (MAP). For example, fusion of an implementation of Okapi BM25 ranking function with Boolean searches for gene names in MeSH fields resulted in 71.5% improvement in MAP over the Okapi ranking function alone and placed third in the 2003 Genomics track evaluation [9].

To cope with the workload growth that outpaces the growth of resources, NLM started the Indexing Initiative project in 1996. This cross-library team is tasked with exploring and implementing indexing methodologies to ensure that MEDLINE and other NLM document collections maintain their quality and currency and thereby contribute to NLM’s mission of maintaining quality access to the biomedical literature.

### NLM medical text indexer

The NLM Medical Text Indexer (MTI) is the main product of the Indexing Initiative and has been providing indexing recommendations based on the MeSH vocabulary since 2002. In 2011, NLM expanded MTI’s role by designating a select set of journals where MTI performs particularly well as MTI first-line (MTIFL) journals. The initial list of 14 MTIFL journals has grown to include 230 journals in 2014. In 2014, MeSH on Demand [10] was developed in collaboration with the NLM MeSH Section providing a simplified user interface to MTI. In its first full month of operation, the interface provided MeSH-based key terms for 140,940 English text documents submitted to it. MTI was also used on a regular basis between 2002 and 2012 to provide fully-automated keyword indexing for NLM’s Gateway [11] meeting abstract collection, which was not manually indexed.

MTI produces semi-automated indexing recommendations based on the MeSH controlled vocabulary and is in daily use to assist Indexers, Catalogers, and NLM’s History of Medicine Division (HMD) in their subject analysis efforts. Although mainly used in indexing efforts for processing MEDLINE citations [12] consisting of identifier, title, and abstract, MTI is also capable of processing arbitrary text, which is the primary mode of text processed by the new MeSH on Demand interface. MTI provides an ordered list of MeSH Main Headings, Subheadings (MEDLINE processing only), and Check Tags as a final result.

**Fig. 1:**
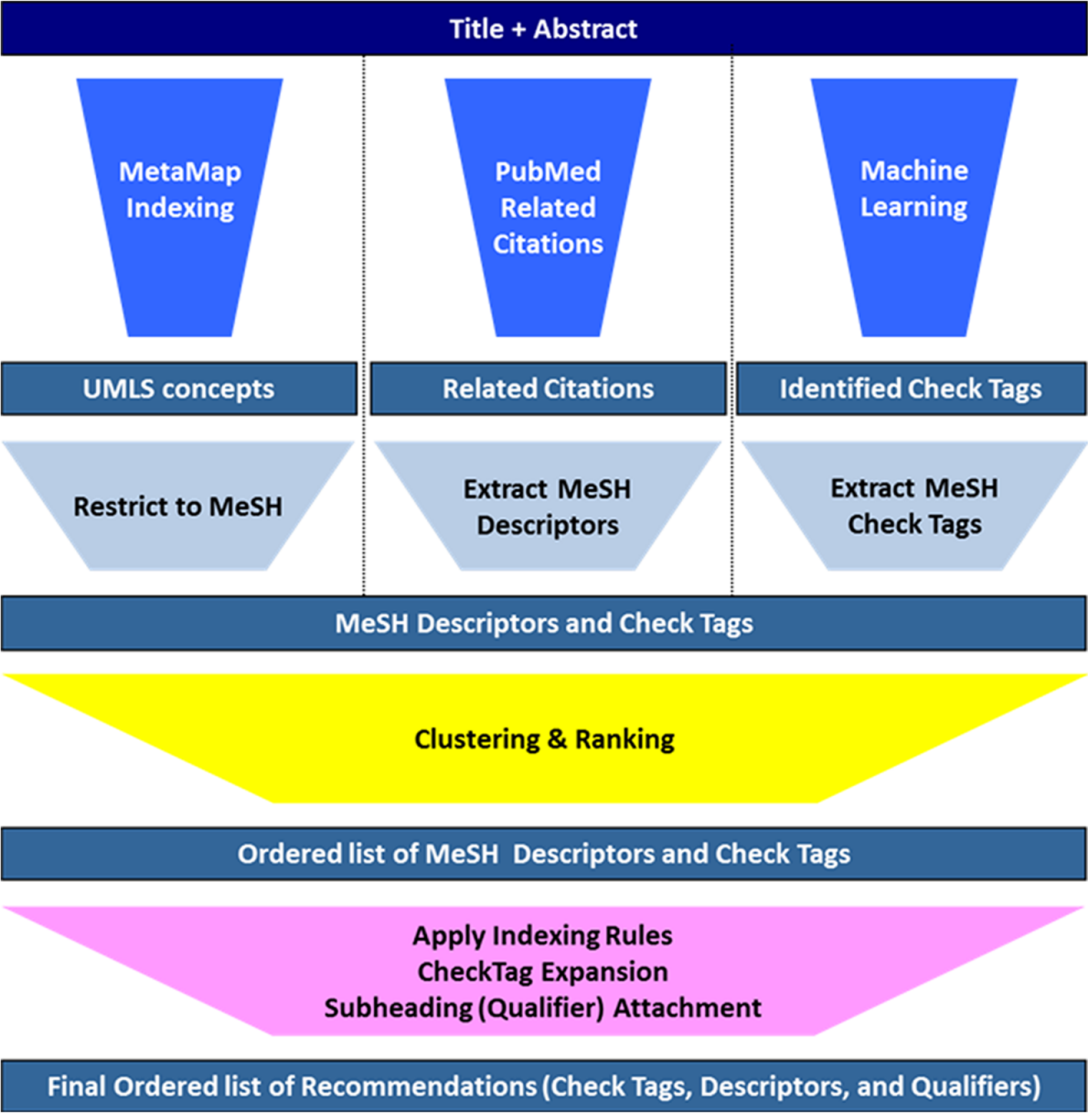
MTI processing flow diagram

The NLM Medical Text Indexer (MTI) [13] combines and ranks terms suggested by three modules depicted in Fig. 1. Figure 1 also shows the logic flow as text is processed through the various components of the MTI system. Each of the major MTI components is very briefly described below.

### MetaMap indexing [14]

A method that applies a ranking function to UMLS Metathesaurus concepts [15] identified by MetaMap [16]. The **Restrict to MeSH** [17] mapping algorithm which finds the closest matching MeSH Heading(s) to a UMLS Metathesaurus concept is used by MTI to map the UMLS Metathesaurus concepts identified by MetaMap Indexing to the required MeSH Descriptors.

### PubMed related citations [18]

The related citations of a document are those documents in the MEDLINE/PubMed database that are the most similar to it. MTI simply requests a list of PubMed Unique Identifiers (PMID) for these related citations that have been indexed and then extracts the MeSH Descriptors from each of the citations.

### Machine learning [19–21]

Twelve of the 40 MeSH Terms listed in Table 1 that MTI considers Check Tags (*Adolescent; Adult; Aged; Aged, 80 and over; Child, Preschool; Female; Humans; Infant; Male; Middle Aged; Swine; and Young Adult*) are reliably (correct 80.62% of the time) identified using a machine learning algorithm that is trained on citations in the MEDLINE database that were indexed in the last three years. These twelve terms used for Machine Learning are highlighted in bold text in Table 1.

**Table 1:** MeSH terms MTI considers check tag

**Adolescent**	History, 18th Century
**Adult**	History, 19th Century
**Aged**	History, 20th Century
**Aged, 80 and over**	History, 21st Century
Animals	History, Ancient
Bees	History, Medieval
Cats	Horses
Cattle	**Humans**
Cercopithecus aethiops	**Infant**
Chick Embryo	Infant, Newborn
Child	**Male**
**Child, Preschool**	Mice
Cricetinae	**Middle Aged**
Dogs	Pregnancy
**Female**	Rabbits
Guinea Pigs	Rats
History of Medicine	Sheep
History, 15th Century	**Swine**
History, 16th Century	United States
History, 17th Century	**Young Adult**

All bolded check tags represent machine learning suggested check tags

Once MTI has the set of ranked lists of MeSH Main Headings produced by the methods described so far, the various lists must be clustered into a single ranked list of recommendations through our **Clustering and Ranking Module** [22]. Once all of the recommendations are ranked and selected, MTI has a post processing feature that validates all of the recommendations and adds or removes select terms based on the targeted end-user. Full end-to-end processing of MEDLINE citations takes approximately 30 - 45 seconds depending on citation length and complexity.

In addition to MEDLINE processing, current uses of MTI where the filtering and results are specifically tuned include **MTI First Line (MTIFL)** and **MeSH on Demand**. The human curation of MTIFL results is called **MTIFL Completion**. MTIFL Completion starts with MTIFL providing the initial indexing for a citation and then a human indexer completes the indexing process by adding any missed terms and removing any incorrect terms provided by MTIFL. The MTIFL Completion citation then goes through the normal manual review process. **MeSH on Demand** [10] is a new use of MTI added in 2014 in collaboration with the NLM MeSH Section. MeSH on Demand is a very simplified interface to the MTI system. The MeSH on Demand interface allows users to provide any text (e.g., MEDLINE citation or free text) as input and provides a list of relevant MeSH Descriptors and MeSH Supplementary Concepts that summarizes the input text and a list of the top ten citations related to the text in PubMed as a result. These results are very heavily filtered in favour of terms with high confidence. Although these new uses of MTI are qualitative indicators of its potential usefulness, the goal of this work is to quantitatively estimate the MTI use and evaluate the quality of its services compared to other available tools. This paper presents our internal log-based evaluation of MTI as well as the results of evaluating MTI in the BioASQ Challenges. Each BioASQ Challenge is a series of challenges on biomedical semantic indexing and question answering with the aim of advancing the state of the art accessibility for researchers and clinicians to biomedical text [23].

## Methods

To answer the questions of whether or not MTI is still useful and relevant, we have used two different approaches evaluating MTI from both an internal and an external viewpoint. We track a large number of statistical markers for MTI on a monthly basis including how every single MeSH Heading is performing, how MTI performs for each journal, how each of the three input methods (MetaMap Indexing, PubMed Related Citations, and Machine Learning) performs individually and in combinations with the two other methods, how often MTI recommendations are referred to by the indexers, and how much MTI is used other than for providing NLM Indexing recommendations.

**Fig. 2:**
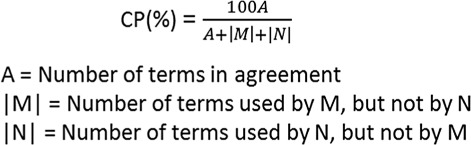
Hooper’s measure of indexing consistency

We used the Hooper Measure of Indexing Consistency [24] shown in Fig. 2, to calculate the consistency percentages for MTI, MTIFL, and previously published indexer consistency studies by Lancaster [25], Leonard [26], Marcetich and Schuyler [27], and Funk and Reid [28]. For the purpose of computing the consistency percentages for MTI and MTIFL, “|N|” is the human indexer and “|M|” is either MTI or MTIFL.

We used the descriptions for the various study categories found in the Funk and Reid [28] paper to correlate the appropriate MTI and MTIFL results to the proper historical study categories. We have also used these descriptions to identify equivalent categories from some of the other historical studies to fill in the results. For example: The definition of the “Descriptors (DESC)” category from Funk and Reid is equivalent to the “Checktags and Main Headings Only” category used in the Lancaster and Leonard studies.

We do not track how well MTI and MTIFL perform when identifying the “Central-concept main headings”, so we were not able include that metric in our evaluation.

For an external evaluation, MTI participated in the “Large-scale online biomedical semantic indexing” task of the 2013 and 2014 BioASQ Challenges [23]. This task is designed to parallel the human indexing currently being done at NLM. During each of the BioASQ Challenges, MTI was impartially and rigorously compared to systems developed by a world-wide community of researchers and industrial teams all performing the same task. We do not consider evaluation of MTI using manual indexing biased because we exclude citations that rely on MTI First Line indexing (MTIFL) from the evaluation and for the citations included in the evaluation MTI recommendations are used at the indexer’s discretion. BioASQ provided us with solid data on how MTI performance compares to other state of the art systems and contributes an outside perspective on MTI. The BioASQ Challenges consisted of three batches of six weekly sets of data to be processed for a total of 18 sets each year. Each data set was processed by the various systems and the results returned to the BioASQ organizers within a 24 h period to make sure none of the citations would have been indexed yet by an indexer which may have biased the results. MTIFL and later default MTI were used as baselines throughout the BioASQ Challenges. A winner was picked for each of the three batches based on the best performing single run of the six possible runs for each batch. So, each BioASQ Challenge had three identified winning systems, one for each of the three batches. Participants were not required to participate in all of the runs during the BioASQ Challenge.

## Results

### Is the NLM medical text indexer used?

**Fig. 3:**
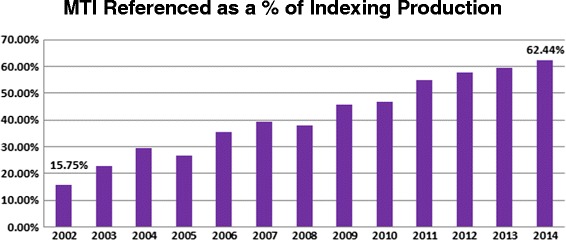
Percentage of indexing production referenced via MTI

**Fig. 4:**
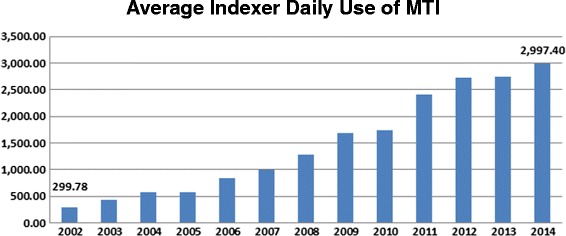
Average daily usage of MTI by indexers

The contract indexers are paid by the article indexed; if they did not feel MTI was useful, they would simply stop referring to the recommendations made by MTI. A recent quote from one of the indexers nicely illustrates the usefulness of MTI: *“…from our perspective, it’s not so much that MTI is STILL useful to the task of indexing, it’s that it is increasingly very useful to the task of indexing …there has been a real shift in perspective on MTI. Indexers used to view it as not helpful …now (most) view it as extremely helpful and overall very accurate”*. Figures 3 and 4 illustrate how daily requests of MTI by the indexers have continually increased from 15.75% of indexing production (299.78 average daily requests) in 2002 to 62.44% of indexing production (2997.40 average daily requests) in 2014, an almost 10-fold increase. This continued and steadily increasing use of MTI by the indexers indicates that they still consider MTI to be useful for their task of indexing.

Another measure of whether or not MTI is useful and relevant is monitoring its use outside of the NLM indexing purposes. Table 2 details the number of MTI requests for 2012, 2013, and 2014 excluding any of our usage. We capture the total number of items: either free text or MEDLINE citations that were processed by MTI; number of MeSH on Demand requests (only available for 2014), and the number of different domains that the web requests come from. These numbers include web requests through our Interactive MTI web page, Batch MTI web page, Web API interface, and the new MeSH on Demand interface. These numbers *do not include* the daily MTI and MTIFL processing of MEDLINE citations, our BioASQ processing, or the testing that is done for the NLM indexing efforts.

**Table 2:** MTI web usage statistics 2012 – 2014

	2012	2013	2014
MTI Requests	44,970	42,919	87,549
# Items processed	3,148,431	7,963,477	11,294,998
MeSH on demand requests	–	–	225,750
# Different domains	118	124	147

A number of outside researchers, authors, and institutions around the world use MTI and MeSH on Demand for various reasons. We do not track who is using our systems or what they are processing, so the only way we know what people are doing with our tools is by interacting with them when there are questions or they need assistance. We know from these interactions that people are using MTI, MTIFL, and MeSH on Demand to identify MeSH keywords for biomedical related course materials, MeSH keywords for their research papers, and to help summarize text they are working with.

### Is the NLM medical text indexer relevant?

**Fig. 5:**
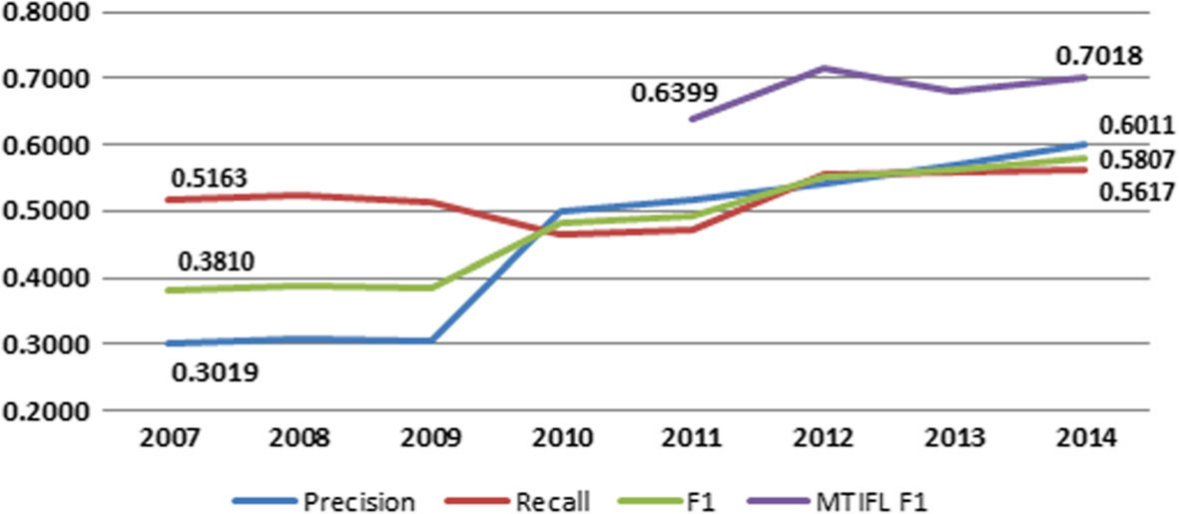
MTI and MTIFL performance 2007 – 2014

We only started tracking MTI performance statistics in 2007. In 2007, MTI statistics showed Precision of 0.3019, Recall of 0.5163, and F_1_ of 0.3810. In 2014, the MTI statistics show significant improvement in Precision and F_1_ with modest gains in Recall reflecting our focus on improving MTI Precision over the years: Precision of 0.6003 (**+0.2992**), Recall of 0.5617 (**+0.0454**), and F_1_ of 0.5807 (**+0.1997**). Figure 5 illustrates the performance changes of MTI between 2007 and 2014 using Precision, Recall, and F_1_ measures. Figure 5 also shows MTIFL F_1_ results between 2011 and 2014. It is clear from Fig. 5 that journals added to the MTIFL program are some of the top performers with the F_1_ score (0.7018) dramatically higher than the overall MTI performance (0.5807).

The MTI statistics for 2014 also show that MTI’s consistency with the human indexers is comparable to the available indexer consistency studies. Table 3 details how both MTI and MTIFL compare with the previously published indexer consistency studies. Table 3 includes information on when each study was performed, how many articles were involved in the study, and where available what percentage of consistency was observed using the Hooper Measure of Indexing Consistency [24]. Each of the included study categories is described below using the Funk and Reid [28] descriptions as a basis and updating the details to conform to today’s MeSH and Indexing practices:

- **Checktags (CT):** Checktags are a special type of MeSH term required to be included for each article and cover species, sex, human age groups, historical periods, pregnancy, and various types of research support (e.g., *Male*).
- **Geographics (GEO):** These are MeSH terms from the Z (Geographicals) MeSH Tree (e.g., *Paris, Indian Ocean*).
- **Descriptors (DESC):** All MeSH terms including Geographicals and Checktags. These were called “Checktags & Main Headings Only” in the Lancaster [25] and Leonard [26] studies.
- **Main headings (MH):** MeSH terms which are not Geographicals or Checktags (e.g., *Lung*).
- **All main headings (no Checktags):** MeSH terms including Geographicals, excluding Checktags.

**Table 3:** Inter-indexer consistency statistics - past and present studies

			Marcetich & Schuyler				
	Lancaster	Leonard	Manual	Computer	Funk & Reid	MTI	MTIFL
Year of study	1968	1975	1981	1981	1983	2014	2014
Number of articles	16	100	50	50	760	673,125	27,068
Checktags (CT)	–	–	–	–	74.70%	62.01%	70.91%
Geographics (GEOG)	–	–	–	–	56.60%	41.52%	57.24%
Descriptors (DESC)	46.10%	48.20%	–	–	55.40%	40.85%	53.97%
Main headings (MH)	–	–	–	–	48.20%	35.17%	48.89%
All main headings (no Checktags)	–	–	39%	43%	–	35.29%	49.12%

The MTI and MTIFL sets in Table 3 include results for all of the citations completed between November 2013 and November 2014 (one standard indexing year). The MTIFL set of 27,068 documents is included in the MTI superset of 673,125 documents.

We also have anecdotal evidence from the NLM Indexing staff stating their feeling is that new indexers are “coming up to speed” and being more productive faster due in part to MTI’s recommendations. The MTI recommendations help new indexers who are not yet as familiar with the entire set of 27,000+ terms in the MeSH Vocabulary as more experienced indexers by providing suggestions they may not be aware of and helping them to limit the scope of terms they might be looking to use. We also have more experienced indexers who rarely, if ever, use MTI recommendations because they are able to index faster without referring to the recommendations.

### External evaluation

MTI was used as the baseline system in the 2013 and 2014 BioASQ Challenges. MTI performed well in both challenges ranking within the top tier teams. Tables 4 and 5 highlight the results of the 2013 and 2014 BioASQ Challenges respectively. The statistics shown in Tables 4 and 5 are unofficial results based on snapshots taken of the BioASQ Results web page on the given dates identified for each table. Tables 4 and 5 both contain the results for the winning team, MTIFL, and MTI. We have included the number of articles completed during each batch, the System Name as provided by the competitors, Precision, Recall, and F_1_ measure for each of the winning systems and the results for both MTI and MTIFL. Please note that the default MTI results were not included as a baseline until the third batch of the 2013 BioASQ Challenge - up to that point we only provided baseline results based on MTIFL filtering.

**Table 4:** 2013 BioASQ results as of October 21, 2013 for winning system and MTI/MTIFL

Batch	# Articles	System name	Precision	Recall	F _1_
1	10,681	System3	0.5602	0.5735	0.5668
		MTIFL	0.5940	0.5196	0.5543
2	11,808	System1	0.5921	0.5670	0.5793
		MTIFL	0.6127	0.5050	0.5537
3	9828	MTI	0.5610	0.6193	0.5887
		MTIFL	0.6027	0.5653	0.5834
		System1	0.5873	0.5760	0.5816

**Table 5:** 2014 BioASQ results as of August 5, 2014 for winning system and MTI/MTIFL

Batch	# Articles	System name	Precision	Recall	F _1_
1	17,061	Asclepius	0.5958	0.5923	0.5941
		MTI	0.5908	0.5614	0.5757
		MTIFL	0.6284	0.5199	0.5690
2	17,073	Antinomyra SYS1	0.6189	0.5863	0.6022
		MTI	0.6012	0.5621	0.5810
		MTIFL	0.6176	0.5367	0.5743
3	18,256	Antinomyra SYS1	0.6527	0.6120	0.6317
		MTI	0.6099	0.5646	0.5864
		MTIFL	0.6400	0.5257	0.5773

In each of the BioASQ Challenges, MTI and MTIFL were very competitive with the winning systems. In 2013, the largest difference in F_1_ between the winning system and MTI/MTIFL was 0.0256 (0.5793 – 0.5537 in batch 2). In 2014, the difference in F_1_ between the winning system and MTI/MTIFL was a little wider at 0.0453 (0.6317 – 0.5864 in batch 3).

## Discussion

The five-fold increase in MTI use by NLM Indexers and the MTI Web Usage statistics detailed in Table 2 provide an indication of how relevant MTI is by showing an increasingly high demand for MTI recommendations. The important thing to note here is that the requests for MTI processing come from researchers, authors, and institutions around the world. For 2014, the data show a significant increase in the number of requests for MTI recommendations and a wider audience of users across more domains. In 2014, we also added a new access point to MTI with the MeSH on Demand interface which is already showing high use. These usage statistics show a sustained and increasing demand for MTI which is a very strong indication that MTI is still relevant.

The MTIFL consistency results in Table 3 (described in the “Results” section) echo the performance gains we see in Fig. 5 when compared to MTI and reflect the fact that only journals where MTI performs very well are added to the MTIFL program. The MTIFL consistency results come close to the Funk and Reid [28] consistency results and the differences may simply reflect the large disparity in the number of articles involved (760 vs 27,068).

MTI and MTIFL performance in the BioASQ Challenges and the fact that both were designated as baselines for the Challenges show that MTI is still relevant.

The benefits of having a challenge like BioASQ pushing systems to improve is evident by how much improvement in performance the winning system, MTI, and MTIFL show over the first BioASQ Challenge. The highest F_1_ measure for a winning system in 2013 was 0.5816 while in 2014 it was increased to 0.6317 (**+0.0501**) [23]. MTI and MTIFL did not show improvement in F_1_, but, did have improvements in Precision from a high of 0.6127 in 2013 to a high of 0.6400 (**+0.0273**) in 2014 reflecting our push to focus on improving Precision over Recall the last few years in both MTI and MTIFL.

The benefits of participating in the 2013 and 2014 BioASQ Challenges for MTI were two-fold:

1. MTI was rigorously and without bias compared to systems developed by a world-wide community of researchers and industrial teams all performing the same task.
2. The challenges provided a forum for the free exchange of methods and ideas allowing the MTI team to incorporate the best practices explored by the participating teams. Incorporating some of these approaches into the MTI workflow in 2013–2014 improved the Precision of MTI indexing suggestions by 4.44% (Recall was improved by 0.08% and F_1_ by 2.23%) [29, 30].

Participating in the BioASQ Challenges also provided us with a renewed interest in machine learning. The 2013 winning system developed by Tsoumakas, et al. [31] was a purely machine learning system. In the past, we ran several experiments [19–21] to see if machine learning might be able to assist MTI and found it to be successful for a handful of MeSH Terms. During our experiments, we ran into problems with unbalanced training sets due to the infrequency of most of the MeSH Terms where we have a very small set of positive examples in comparison to the set of negative examples. In the end, only the results for some of the most frequently used MeSH Terms were viable enough to incorporate into MTI. In the first BioASQ Challenge, we learned that Tsoumakas et al. were able to successfully overcome this problem and performed slightly better than MTI in most of the weekly sets as shown in Table 4 (described in the “Results” section).

Another interesting topic from the BioASQ Challenges that we had not pursued before with MTI but which proved beneficial in the BioASQ Challenges was a learning-to-rank method used by Mao and Lu [32, 33]. Our analysis of the MTI recommendations not provided to the indexers shows that MTI incorrectly assigns low scores and removes many of the actual indexing terms used by the human indexers. The learning-to-rank algorithms seem to identify these abandoned and ignored terms allowing the system to move them up higher in the ranked list. In fact Mao and Lu used the MTI results as one of their features in their approach.

The winning system in the second and third batches of the 2014 BioASQ Challenge (Antinomyra) was developed by Liu et al. [34], their system combines the support vector machines explored by Tsoumakas et al. [31] and the learning-to-rank approach by Mao and Lu [32, 33] into a system that outperformed either approach individually as shown in Table 5 (described in the “Results” section).

**Fig. 6:**
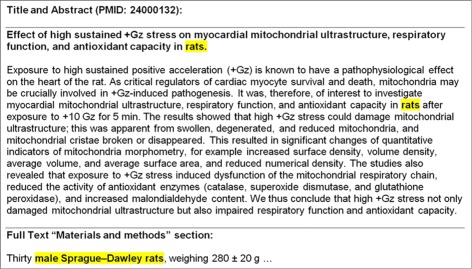
Title and abstract versus full text example (PMID: 24000132)

Competing in the BioASQ Challenges also provided the impetus for us to explore why MTI was missing some of the terms that the human indexers use. The main reason we found for missing the most frequently occurring MeSH Terms (Check Tags) was that the necessary information was contained in the full text available to indexers, but not in the Title or Abstract that MTI was using to compute its recommendations. This specific information tends to be found in the “Methods” section of the full text where the authors describe how their experiments were structured. Usually this is where we see information on the type of experiment subjects (*Animal, Humans*, or both), sex of the subjects (*Male* or *Female*), age of the subjects (*Infant, Newborn; Infant; Child, Preschool; Child; Adolescent; Young Adult; Adult; Middle Aged; Aged;* and *Aged, 80 and over*), and if an Animal study, what kind of animals (*Mice, Rats, Hamsters,* etc.). A simple example of this can be seen in Fig. 6 where we have highlighted the descriptions of the experiment subjects in the Title, Abstract, and Full Text. For PMID 24000132, Fig. 6 illustrates how the author provided only a very general description of “rats” for the experiment subjects in the Title and Abstract and nothing about what sex the rats were, or what specific type of rats they were. The full text on the other hand includes very specific information in the “Methods” section of the paper letting us know the subjects were “Male” “Sprague-Daley rats” in the experiment. This information from the full text is critical to MTI because recommending just *Rats* would only provide one-third of the correct answer. The human indexer would use *Male*, *Rats*, and *Rats, Sprague-Dawley*.

### Future work

We are currently looking at several ways to incorporate machine learning and learning-to-rank either into MTI, or as a starting point for a next generation MTI.

One very promising approach we are investigating is to use Wilbur and Kim’s Stochastic Gradient Descent approach [35] as a starting point for a next generation MTI and then add in lookup lists, machine learning, indexing rules, and filtering from the existing MTI system. The preliminary indications are encouraging showing that the two systems are in fact complementary.

Mao and Lu [32, 33] are also seeing very good results with their learning-to-rank algorithm which uses MTI as one of the features. We are currently working with them to see if MTI can use their ranking results to try to salvage some of the abandoned MTI recommendations.

We intend to start working with full text (e.g., from PubMed Central) to see if we can improve MTI performance with a focused look at the full text. Only 10% of the articles MTI processes have XML full text in PubMed Central, but it would provide us with data to explore full text.

MTI is also being considered to possibly expand its role by assisting with mapping OLDMEDLINE [36] terms to the latest version of the MeSH Vocabulary for citations originally printed in hardcopy indexes published prior to 1966, and the possibility of providing keywords for citations that normally would not be humanly indexed to provide additional access points that would assist in retrieval.

## Conclusion

After twelve years and two BioASQ Challenges it was a perfect time to look around and perform a reality check to determine if MTI was indeed still useful and relevant. In this paper we have presented several qualitative and quantitative reasons why we think that MTI is in fact still useful and relevant. The statistics on how much MTI is used by the indexers and by people outside of the US National Library of Medicine show that MTI usage continues to grow. The unbiased external review of MTI by the BioASQ Challenges where MTI provided two of the baseline systems showed us that MTI is still one of the benchmarks for biomedical semantic indexing; but it also proved that we have room for improvement, and even provided possible research avenues to make some of those improvements to MTI. For the first time, the BioASQ Challenges also provided us with a third-party mechanism to compare MTI against other world-class systems in an unbiased and principled manner.

## Abbreviations

CT: MeSH check tag
MeSH: Medical Subject Headings
MH: MeSH main headings, also described as descriptors
HMD: US National Library of Medicine History of Medicine Division
MTI: NLM medical text indexer
MTIFL: NLM Medical text indexer first line
NLM: US National Library of Medicine
PMID: PubMed unique identifier
SH: MeSH subheadings, also described as qualifiers

## References

[1] Fact sheet: medical subject headings (mesh). http://www.nlm.nih.gov/pubs/factsheets/mesh.html. Accessed 27 June 2016.

[2] Medline indexing online training course - check tags. https://www.nlm.nih.gov/bsd/indexing/training/CHK_010.html. Accessed 13 Feb 2017.

[3] Faq: index medicus chronology. https://www.nlm.nih.gov/services/indexmedicus.html. Accessed 2 Dec 2016.

[4] Pubmed. https://www.ncbi.nlm.nih.gov/pubmed. Accessed 2 Dec 2016.

[5] Pubmed tutorial: automatic term mapping. https://www.nlm.nih.gov/bsd/disted/pubmedtutorial/020_040.html. Accessed 2 Dec 2016.

[6] Pubmed help. https://www.ncbi.nlm.nih.gov/books/nbk3827/#_pubmedhelp_filters_. Accessed 2 Dec 2016.

[7] Hersch W, Voorhees E (2009). Trec genomics special issue overview. Inf Retr.

[8] Roberts K, Simpson M, Demner-Fushman D, Voorhees E, Hersh W (2016). State-of-the-art in biomedical literature retrieval for clinical cases: a survey of the trec 2014 cds track. Inf Retr.

[9] Yeung D, Clarke C, Cormack G, Lynam T, Terra E. Task-specific query expansion (multitext experiments for trec 2003). NIST Special Publication: SP 500–255 Proceedings The Twelfth Text Retrieval Conference (TREC 2003), 2003;810–19.

[10] Mesh on demand homepage. https://www.nlm.nih.gov/mesh/MeSHonDemand.html. Accessed 13 Feb 2017.

[11] Fact sheet: Nlm gateway. https://www.nlm.nih.gov/pubs/factsheets/gateway.html. Accessed 27 June 2016.

[12] Medline/pubmed data element (field) descriptions. https://www.nlm.nih.gov/bsd/mms/medlineelements.html. Accessed 27 June 2016.

[13] Mork J, Jimeno Yepes A, Aronson A. The nlm medical text indexer system for indexing biomedical literature. 2013. BioASQ Workshop. Valencia, Spain, September 2013. BioASQ - http://bioasq.org/sites/default/files/Mork.pdf.

[14] Aronson A. The mmi ranking function whitepaper (1997). http://ii.nlm.nih.gov/Publications/Papers/ranking.pdf. Accessed 13 Feb 2017.

[15] Fact sheet: Umls metathesaurus. https://www.nlm.nih.gov/pubs/factsheets/umlsmeta.html. Accessed 27 June 2016.

[16] Aronson A, Lang F (2010). An overview of metamap: Historical perspective and recent advances. J Am Med Inform Assoc.

[17] Bodenreider O, Nelson S, Hole W, H C. Beyond synonymy: Exploiting the umls semantics in mapping vocabularies. Proc AMIA Symp. 1998;:815–9. PMC2232139 9929332

[18] Lin J, Wilbur W (2007). Pubmed related articles: a probabilistic topic-based model for content similarity. BMC Bioinforma.

[19] Jimeno-Yepes A, Mork J, Demner-Fushman D, Aronson A. Automatic algorithm selection for mesh heading indexing based on meta-learning. International Symposium on Languages in Biology and Medicine. 2011. https://ii.nlm.nih.gov/Publications/Papers/Antonio_MTI_ISLBM_2011.pdf.

[20] Jimeno-Yepes A, Wilkowski B, Mork J, Demner-Fushman D, Aronson A. Medline mesh indexing: Lessons learned from machine learning and future directions. IHI ’12 Proceedings of the 2nd ACM SIGHIT International Health Informatics Symposium. 2012;:737–42. http://dl.acm.org/citation.cfm?id=2110450.

[21] Jimeno-Yepes A, Mork J, Demner-Fushman D, Aronson A (2012). A one-size-fits-all indexing method does not exist: automatic selection based on meta-learning. JCSE.

[22] Mti clustering and ranking process. https://ii.nlm.nih.gov/MTI/Details/cluster.shtml. Accessed 13 Feb 2017.

[23] Tsatsaronis G, Balikas G, Malakasiotis P, Partalas I, Zschunke M, Alvers M, Weissenborn D, Krithara A, Petridis S, Polychronopoulos D, Almirantis Y, Pavlopoulos J, Baskiotis N, Gallinari P, Artiéres T, Ngomo A, Heino N, Gaussier E, Barrio-Alvers L, Schroeder M, Androutsopoulos I, Paliouras G (2015). An overview of the bioasq large-scale biomedical semantic indexing and question answering competition. BMC Bioinforma.

[24] Hooper R. Indexer consistency tests: origin, measurement, results, and utilization: Bethesda, Md.: IBM Corporation; 1965. (TR95-56).

[25] Lancaster F (1968). Evaluation of the medlars demand search service.

[26] Leonard L (1975). Inter-indexer consistency and retrieval effectiveness: measurement of relationships. Ph.D. thesis.

[27] Marcetich J, Schuyler P. The use of aid to promote indexing consistency at the national library of medicine. Eighty-first Annual Meeting of the Medical Library Association.

[28] Funk M, Reid C (1983). Indexing consistency in medline. Bull Med Libr Assoc.

[29] Mork J, Demner-Fushman D, Schmidt S, Aronson A. Recent enhancements to the nlm medical text indexer. In: CLEF2014 Working Notes: Working Notes for CLEF 2014 Conference. Sheffield, UK, September 15–18, 2014;1180:1328–36. http://ceur-ws.org/Vol-1180/CLEF2014wn-QA-MorkEt2014.pdf.

[30] Mork J, Demner-Fushman D, Schmidt S, Aronson A (2014). Vocabulary density method for customized indexing of medline journals. Poster AMIA.

[31] Tsoumakas G, Laliotis M, Markantonatos N, Vlahavas I. Large-scale semantic indexing of biomedical publications at bioasq. A Post-Conference Workshop of Conference and Labs of the Evaluation Forum 2013 (CLEF 2013) Valencia, Spain, September 27th, 2013;1094. http://ceur-ws.org/Vol-1094/bioasq2013_submission_6.pdf.

[32] Mao Y, Lu Z. Ncbi at the 2013 bioasq challenge task: Learning to rank for automatic mesh indexing. http://bioasq.org/sites/default/files/2013_Mao_Lu_NCBI_Methodology.pdf.

[33] Mao Y, Wei C, Lu Z. Ncbi at the 2014 bioasq challenge task: Large-scale biomedical semantic indexing and question answering. CLEF2014 Working Notes: Working Notes for CLEF 2014 Conference. Sheffield, UK, September 15–18. 2014;1180:1319–27. http://ceur-ws.org/Vol-1180/CLEF2014wn-QA-MaoEt2014.pdf.

[34] Liu K, Wu J, Peng S, Zhai C, Zhu S. The fudan-uiuc participation in the bioasq challenge task 2a: The antinomyra system. CLEF2014 Working Notes: Working Notes for CLEF 2014 Conference. Sheffield, UK, September 15–18, 2014;1180:1311–18. http://ceur-ws.org/Vol-1180/CLEF2014wn-QA-LiuEt2014.pdf.

[35] Wilbur W, Kim W (2014). Stochastic gradient descent and the prediction of mesh for pubmed records. AMIA.

[36] Oldmedline data description. https://www.nlm.nih.gov/databases/databases_oldmedline.html. Accessed 7 June 2016.

